# Impacts of impaired mitochondrial dynamics in hearing loss: Potential therapeutic targets

**DOI:** 10.3389/fnins.2022.998507

**Published:** 2022-10-05

**Authors:** Tianyuan Zou, Bin Ye, Kaili Chen, Andi Zhang, Dongye Guo, Yi Pan, Rui Ding, Haixia Hu, Xingmei Sun, Mingliang Xiang

**Affiliations:** ^1^Department of Otolaryngology and Head and Neck Surgery, Ruijin Hospital, Shanghai Jiao Tong University School of Medicine, Shanghai, China; ^2^Ear Institute, Shanghai Jiao Tong University School of Medicine, Shanghai, China; ^3^Shanghai Key Laboratory of Translational Medicine on Ear and Nose Diseases, Shanghai, China

**Keywords:** mitochondrial dynamics, cochlea, hearing loss, OPA1, mitochondrial quality control

## Abstract

Mitochondria are the powerhouse of the cells. Under physiological conditions, mitochondrial fission and fusion maintain a dynamic equilibrium in the cytoplasm, which is referred to as mitochondrial dynamics. As an important approach to regulating mitochondrial function and quantity, the role of mitochondrial dynamics has been demonstrated in the pathogenesis of various disease models, including brain damage, neurodegeneration, and stress. As the vital organ of the peripheral auditory system, the cochlea consumes a significant amount of energy, and the maintenance of mitochondrial homeostasis is essential for the cochlear auditory capacity. *OPA1* functions as both a necessary gene regulating mitochondrial fusion and a pathogenic gene responsible for auditory neuropathy, suggesting that an imbalance in mitochondrial dynamics may play a critical role in hearing loss, but relevant studies are few. In this review, we summarize recent evidence regarding the role of mitochondrial dynamics in the pathogenesis of noise-induced hearing loss (NIHL), drug-induced hearing loss, hereditary hearing loss, and age-related hearing loss. The impacts of impaired mitochondrial dynamics on hearing loss are discussed, and the potential of mitochondrial dynamics for the prevention and treatment of hearing loss is considered.

## Mitochondrial dynamics

**FIGURE 1:**
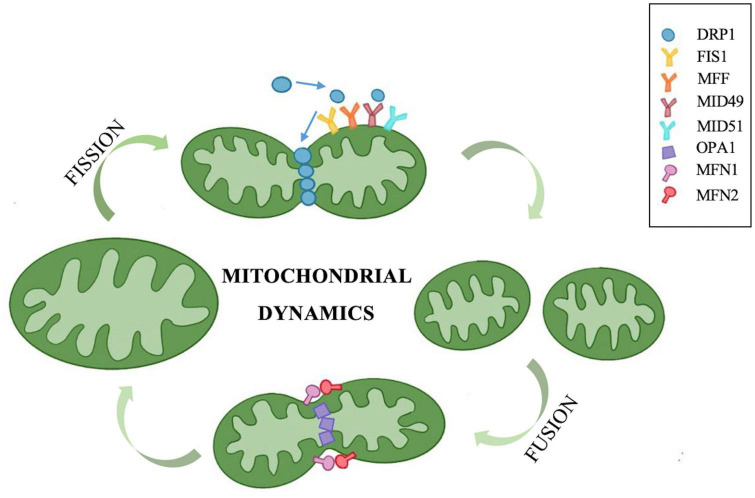
Mitochondrial fission and fusion maintain a dynamic equilibrium *via* a series of molecules. The GTPase DRP1 located in the cytoplasm plays a dominant role in mitochondrial fission. The DRP1 receptor proteins FIS1, MFF, and MID49/51 are all distributed in the outer mitochondrial membrane. These receptor proteins can recruit DRP1 to the outer membrane to form a helical structure that hydrolyzes GTP, induces mitochondrial constriction and severing, and promotes mitochondrial fission. OPA1, MFN1, and MFN2 are three types of GTPases that govern mitochondrial fusion. MFN1 and MFN2 are localized in the outer mitochondrial membrane and form homologous or heterologous complexes that mediate mitochondrial coupling. OPA1 is involved in inner mitochondrial membrane fusion and the connection of cristae remodeling, and OPA1 mediates inner membrane fusion in turn after outer membrane fusion.

Mitochondria are highly dynamic organelles found in eukaryotic cells, with a double-membrane structure composed of the outer mitochondrial membrane (OMM), mitochondrial intermembrane space, inner mitochondrial membrane (IMM), and mitochondrial matrix (Chan, 2020). Under physiological conditions, mitochondrial fission and fusion maintain a dynamic equilibrium and regulate cellular activity *via* network structure homeostasis (Figure 1). The dysregulation of mitochondrial fission and fusion is an abnormal mitochondrial dynamic that results in a dramatic increase or decrease in mitochondrial numbers, increased mitochondrial size or fragmentation, and an uneven distribution of mitochondria (Chen et al., 2014). Elongated tubular structures appear when mitochondrial fusion increases or fission decreases, and granular structures appear when fusion decreases or fission increases. With the transduction of apoptotic signals, mitochondrial fission increases, and mitochondrial morphology undergoes a shift to globules. At the same time, OMM permeability, structural instability of mitochondrial cristae, and the subsequent release of reactive oxygen species (ROS) and cytochrome C increase, which then activate multiple endogenous apoptotic pathways and accelerate cell death (Youle and Karbowski, 2005). This disruption of the equilibrium of mitochondrial dynamics alters mitochondrial structure, morphology and function, and also impacts cellular metabolism, motility, division, mitophagy and apoptosis, particularly in cells with high energy consumption, such as cochlear auditory cells, neurons, and cardiomyocytes.

### Mitochondrial fission and its regulation

Mitochondrial fission can generate mitochondrial fragments, increase the number of mitochondria, facilitate mitochondrial movement and division, promote mitophagy to remove damaged mitochondria, and regulate mitochondrial quantity and quality (Chan, 2020). Excessive mitochondrial fission can cause increased OMM permeability and the release of apoptosis-triggering factors, such as ROS, resulting in apoptosis. Currently, the proteins related to mitochondrial fission mainly include mitochondrial dynamin-related protein 1 (DRP1), mitochondrial fission protein 1 (FIS1), mitochondrial fission factor (MFF), and mitochondrial dynamics protein 49/51 (MID49/51) (El-Hattab et al., 2018). Among these, DRP1 plays a dominant role. As a GTPase, DRP1 is primarily located in the cytoplasm. After activation, it localizes to the DRP1 receptor in mitochondria, forming an annular lariat structure and fission focal point, thus causing mitochondria to shrink and divide. This is an essential initial step for mitochondrial fission. The DRP1 receptor proteins FIS1, MFF, and MID49/51 are all distributed in the OMM. These receptor proteins can recruit DRP1 to the OMM to form a helical structure that hydrolyzes GTP, induces mitochondrial constriction and severing, and promotes mitochondrial fission (Youle and Karbowski, 2005).

### Mitochondrial fusion and its regulation

Mitochondrial fusion produces either tubular or elongated mitochondria, which are interconnected to form a dynamic network that increases the interconnections between them (Tilokani et al., 2018). Energy transport, signal exchange, and mitochondrial DNA (mtDNA) complementation between mitochondria are facilitated by this network. Insufficient mitochondrial fusion can result in increased mitochondrial fragmentation and apoptosis. Optic atrophy 1 (OPA1), mitochondrial fusion-related protein 1 (mitofusin 1, MFN1), and mitochondrial fusion-related protein 2 (mitofusin 2, MFN2) are three types of GTPases that govern mitochondrial fusion. MFN1 and MFN2 are localized in the OMM and form homologous or heterologous complexes that mediate mitochondrial coupling. MFN1 has a stronger hydrolytic activity and fusion efficiency, while MFN2 plays a crucial role in the regulation of Ca^2+^ homeostasis. Cells lacking either MFN1 or MFN2 tend to undergo mitochondrial fragmentation, and the mitochondrial shape presents as granules, while the deficiency of both will result in the loss of fusion function, which produces more severe mitochondrial fragmentation and functional impairment (Gao and Hu, 2021). OPA1 is involved in IMM fusion and the connection of cristae remodeling, and OPA1 mediates IMM fusion in turn after OMM fusion. Furthermore, the abundance and activity of mitochondrial fusion proteins are proportional to the content of adenosine triphosphate (ATP). Abnormal fusion of cellular mitochondria leads to impaired oxidative phosphorylation, increased ROS production, and mtDNA loss (Chen and Chan, 2010).

## Abnormal mitochondrial dynamics and hearing loss

**FIGURE 2:**
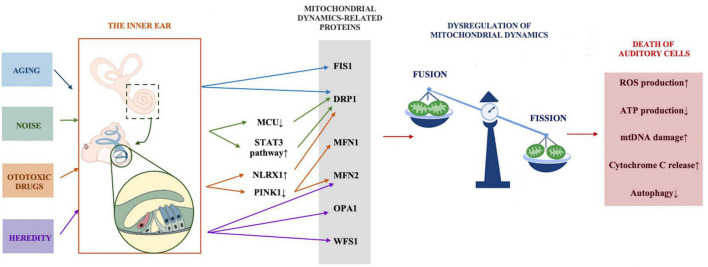
The possible role of abnormal mitochondrial dynamics in common forms of hearing loss. Stress conditions including aging, noise, ototoxic drugs, and heredity act on mitochondrial dynamics regulation-related proteins through various mechanisms, mitochondrial dynamics tend to be in the state of fission, excessive fission leads to increased ROS and cytochrome C production, reduced ATP production, mtDNA damage and the inhibition of autophagy, and eventually results in the death of auditory cells.

Different hearing loss models have verified the effect of mitochondrial dysfunction on auditory function. The accumulation of mtDNA mutations leads to presbycusis (Kim et al., 2019), mitochondrial *12SrRNA* gene A1555G mutation results in non-syndromic hereditary hearing loss and susceptibility to ototoxic drug aminoglycoside antibiotics (Ding et al., 2013), and noise causes mitochondria to produce an excessive amount of ROS and promotes the death of cochlear hair cells. An increasing number of audiology researchers are interested in the restoration of mitochondrial function or quantity as an important means to restore hearing capacity. In recent years, abnormal mitochondrial dynamics have been found to play critical roles in the onset and progression of many neurodegenerative diseases, including Alzheimer’s disease (AD) and Parkinson’s disease (PD), and ameliorating dysfunctional mitochondrial dynamics to restore mitochondrial function can alleviate the deterioration caused by neurodegenerative diseases (Nunnari and Suomalainen, 2012). Since mitochondrial dynamics have been rarely explored in the field of hearing, we propose the possible role of abnormal mitochondrial dynamics in common forms of hearing loss to better understand its association with hearing loss (Figure 2).

### Abnormal mitochondrial dynamics in noise-induced hearing loss

Noise is one of the most important environmental factors that cause hearing loss, and it can be divided into three categories: occupational noise, environmental noise, and recreational noise (Basner et al., 2014). According to the 2021 WHO World Hearing Report, recreational noise can leave 50% of young people (12–35 years old) at risk of hearing loss (World Health Organization [WHO], 2021). Therefore, noise-induced hearing loss (NIHL) poses a significant threat to the hearing of young people (Chadha et al., 2021). Studies have shown that exposure to mild or moderate levels of noise can lead to temporary hearing threshold shift (TTS) which is reversible. With the increase of noise intensity or the extension of exposure time, TTS turns into a permanent threshold shift (PTS). Cochlear hair cells suffer permanent damage and loss, and the hearing threshold cannot be restored (Hong et al., 2013). Among the many hypotheses about the mechanism of NIHL, oxidative stress damage brought on by the excessive production of ROS is generally acknowledged. Mitochondria are the primary generator of ROS in mammals (Fetoni et al., 2019). Recent studies suggest that noise may induce the mitochondria in auditory cells to enter a state of excessive division, resulting in mitochondrial fragmentation, reduced ATP synthesis, and increased levels of ROS. Increasing mitochondrial fusion could be an effective treatment for noise-induced deafness.

Mitochondrial Ca^2+^ uniporter (MCU), a transmembrane protein for unidirectional Ca^2+^ absorption by mitochondria, is the major channel of mitochondrial Ca^2+^ uptake and is critical for maintaining mitochondrial calcium homeostasis (Alevriadou et al., 2021). A study in 2021 (Manikandan et al., 2021) discovered that knocking out *Mcu* in FVB/NJ mice has no effect on hearing or cochlear development. However, significantly elevated auditory brainstem response (ABR) thresholds were detected 3 weeks after birth at a frequency of 32 kHZ, and by 4 weeks after birth, the thresholds of all frequencies had increased by about 20–40 dB. These results demonstrate that the *Mcu* gene is essential for maintaining normal hearing in mice. After noise exposure, the immunostaining intensity of MCU in the cochlear hair cells of CBA/J mice was significantly enhanced. Both the intra-tympanic delivery of MCU siRNA and the intra-peritoneal injection of specific MCU inhibitor Ru360 can alleviate cochlear hair cells loss and NIHL (Wang et al., 2019). It has also been reported that in the vascular smooth muscle cells (VSMCs) of *Mcu* knockout mice, the phosphorylation level of DRP1, the key regulator of mitochondrial fission, is significantly higher than in WT littermates; their mitochondrial ATP content is also reduced and cells tend to divide, thereby inhibiting cell proliferation (Koval et al., 2019). Therefore, we speculate that enhanced mitochondrial DRP1 activity in the cochlea of *Mcu* knockout mice leads to a tendency for increasing mitochondrial fission, resulting in a decrease in mitochondrial ATP content and an increase in ROS. From the above articles, we speculate that noise stress prompts the cochlear mitochondria to further consume ATP and generate ROS, resulting in an insufficient mitochondrial energy supply and increased cellular oxidative stress, which in turn leads to the loss of cochlear hair cells.

A recent study from the University of Maryland School of Medicine (Milon et al., 2021) integrated the results of the single-cell sequencing of various cell types in the whole cochlea and discovered that the immune response mediated by the signal transducer and activator of transcription 3/interferon regulatory factor 7 (STAT3/IRF7) pathway plays an important role in the early stages of noise-induced deafness in mice. Wilson et al. (2014) reported in 2014 that intraperitoneal administration of STAT3 inhibitor Cucurbitacin I in male CBA/CaJ mice before noise exposure can inhibit ROS formation in cochlear outer hair cells (OHCs), thus protecting OHCs and drastically reducing their ABR and distortion product otoacoustic emission (DPOAE) thresholds. However, the authors did not explain why the inhibition of STAT3 reduces ROS production in OHCs. In 2019, Zhou et al. (2019) found, in a mouse model of cerebral ischemia injury, that activation of the STAT3 pathway in microglia recruits DRP1 on the mitochondrial surface and increases mitochondrial fission. An imbalance in mitochondrial dynamics promotes increased ROS levels and cell death in microglia. By inhibiting the STAT3 pathway, mitochondrial fission can be reduced and the release of ROS can be inhibited, thus promoting the survival of microglia. Therefore, we hypothesize that inhibiting the STAT3 pathway may reduce hearing loss in NIHL by inhibiting the motochondrial fission protein DRP1 in cochlear hair cells, thereby reducing mitochondrial fission and ROS production, and thus promoting hair cell survival and hearing protection.

### Abnormal mitochondrial dynamics and ototoxic drug-induced hearing loss

Similar to other sensory nervous systems, the auditory system is susceptible to damage from ototoxic drugs, which can result in mitochondrial dynamics dysfunction. Common ototoxic drugs include aminoglycoside antibiotics, platinum chemotherapy drugs, and diuretics. As a member of the nucleotide-binding domain and leucine-rich repeat-containing (NLRs) family, NLR family member X1 (NLRX1) localizes to mitochondria and is mainly expressed in tissues and organs with a high energy metabolism. NLRX1 was found in the cytoplasm of inner hair cells (IHCs) and OHCs in the cochlea of C57BL/6 mice, particularly in the ciliated region. The level of NLRX1 increases with the growth and development of C57BL/6 mice. The level of expression in cochlear hair cells peaks at the age of 3 months, then declines with aging at 9 months. The levels of NLRX1 and the apoptotic factors p-c-Jun N-terminal kinase (p-JNK) and caspase3 were significantly elevated in a 3-month-old mouse model of hearing loss induced by neomycin (Yang et al., 2016). This experiment indicated that NLRX1 is involved in neomycin-induced cochlear hair cell apoptosis and hearing loss in C57BL/6 mice. In another experiment, rotenone (a mitochondrial respiratory chain inhibitor) was added to N2a neural cell lines in which the *Nlrx1* gene was either knocked out or overexpressed, which demonstrated that *Nlrx1* knockout cells could inhibit rotenone-induced apoptosis, whereas cells that overexpress *Nlrx1* had significantly elevated phospho-DRP1, which promoted mitochondrial fission. Subsequent electron microscopy revealed that the number of mitochondria had increased dramatically, but the mitochondrial cristae structure had swollen significantly, indicating that mitochondrial function had been severely compromised after *Nlrx1* was upregulated (Imbeault et al., 2014). We speculate that in neomycin-induced mouse cochlear hair cells, the increase of NLRX1 protein results in the phosphorylation of the motochondrial fission protein DRP1, which leads to the increase of phospho-DRP1, which in turn promotes mitochondrial fission and the disruption of mitochondrial cristae structures, ultimately leading to the death of auditory cells.

Platinum-based chemotherapy drugs are the primary pharmaceutical used for the clinical treatment of solid tumors, especially in children (Oun et al., 2018). The major side effect is irreversible hearing loss. Phosphatase and tensin homolog (PTEN)-induced kinase 1 (PINK1) is a serine/threonine-protein kinase that is primarily expressed in the OMM and acts as a molecular sensor for mitochondrial quality control. PINK1 continuously monitors mitochondrial status, identifying damaged mitochondria and removing them through selective mitophagy, thus ensuring the health and function of mitochondria. Conversely, abnormal PINK1 expression leads to mitochondrial dysfunction. PINK1 is widely expressed in IHCs, OHCs, spiral ganglion neurons, and the stria vascularis of the mouse cochlea (Oh et al., 2020). The expression of PINK1 decreases first and then increases, which is observed in the hair cell line HEI-OC1 damaged by cisplatin. Inhibiting the expression of PINK1 in the HEI-OC1 cell line by siRNA can reduce the level of autophagy induced by cisplatin and promote apoptosis (Yang et al., 2018). As the pathogenic gene of human familial hereditary Parkinson’s disease, PINK1 is a key initiating molecule that regulates the mitophagy system. In the SH-SY5Y cell of the PD model, Parkin is activated by elevated PINK1 and then transported to the outer membrane, where it catalyzes the ubiquitination and degradation of the fusion protein MFN1/2, causing mitochondria to divide excessively, thus increasing autophagy and cell survival (Gegg et al., 2010). Thus, it is evident that mitochondrial dynamics and mitophagy are inextricably related and work together to maintain mitochondrial homeostasis in cells. When hair cells are damaged by cisplatin, reduced PINK1 will lead to insufficient fission of damaged mitochondria and inhibit the process of autophagy, thereby triggering cell death.

### Abnormal mitochondrial dynamics in the progression of presbycusis

The world now has an expanding and aging society as medical standards and quality of life improve, especially in China. According to the 7th national population census released by the National Bureau of Statistics of China, as of November 1, 2020, the number of people over 60 years old in China reached 264 million, or approximately 18.7% of the total population (Tu et al., 2021). The share of the population above 65 accounted for 13.5% of the total, or 190 million people. It is speculated that by 2025, there will be over 300 million elderly people in China; at that point, they will comprise over 20% of the population, and the country will enter a super-aging-society state (Tu et al., 2021). Presbycusis is the most common sensory neurological disorder, which greatly affects a person’s quality of life and physical health.

Enlarged and elongated mitochondria are found in many aging human tissues, but the mechanism of their generation remains unclear. Yoon et al. (2006) found that a high percentage of mitochondria were enlarged or elongated in H_2_O_2_-induced aging human diploid fibroblast (HDFs) cells. At the same time, the presence of the mitochondrial fission protein FIS1 was significantly reduced. Overexpression of FIS1 could drastically reverse both mitochondrial elongation and cellular senescence. This study suggests that aging, oversized mitochondria are closely related to abnormal mitochondrial dynamics. Addressing disorders of mitochondrial dynamics can extend the lifespan of cells, and the regulation of mitochondrial dynamics may be a key to preventing aging. Mitochondrial dysfunction is an important mechanism of human aging and presbycusis. Abnormal mitochondrial dynamics may be involved in the development of presbycusis. P43 is a 43 kDa protein located in the mitochondria, and is the triiodothyronine (T3) thyroxine l receptor synthesized by the thyroid hormone receptor alpha (*Thr*α) gene. It has been reported that the hearing ability of 1-month-old *p43* knockout mice is comparable to that of WT mice. However, at 6 months of age, the ABR threshold of *p43*^–/–^ mice at 4–32 kHz is significantly elevated. By counting hair cells, a significant loss of the OHCs was observed in the apical, middle, and basal cochlear regions of *p43*^–/–^ mice. These results suggest that *p43* knockout leads to the early onset of presbycusis in mice. Subsequent transmission electron microscope investigations found that the mitochondrial size in OHCs of *p43*^–/–^ mice was significantly larger, indicating that abnormal mitochondrial dynamics may be involved (Affortit et al., 2021).

Researchers have also discovered that the mitochondrial fission protein DRP1 and mitophagy levels were both reduced in hydrogen peroxide-induced aging models of the HEI-OC1 cell line and cochlear explants (Lin et al., 2019). In this experiment, 8-month-old C57BL/6 mice were intraperitoneally injected with the DRP1 inhibitor midiv-1 every 3 days for 4 months. Compared with normal 12-month-old mice, mice receiving midiv-1 injections had decreased mitochondrial ATP synthesis, elevated ABR thresholds, and increased hair cell loss, indicating that DRP1 inhibition inhibited mitochondrial fission, thereby aggravating the occurrence of presbycusis. These studies have shown that the aging cochlea exhibits morphological mitochondrial damage and a loss of energy production, as well as that mitochondrial fission is likely to be implicated in the aging cochlea and the development of presbycusis.

### Abnormal mitochondrial dynamics as a potential mechanism underlying hereditary hearing loss

Hereditary hearing loss accounts for more than half of congenital hearing loss, of which about 70% are non-syndromic deafness (Schrijver, 2004). The mitochondrial *12SrRNA* gene mutation accounts for about 3% of the cases of non-syndromic deafness, and it is also one of the three most common pathogenic genes for non-syndromic deafness in the Chinese population. Recent studies suggest that the methyltransferase mitochondrial transcription factor B1 (mtTFB1) disrupts ribosomal function by increasing the methylation level of mitochondrial *12SrRNA*, thus inducing hearing loss (Raimundo et al., 2012). An abnormal methylation level of mitochondrial DNA elicits abnormal mitochondrial dynamics, causing mitochondria to split and fragment, which eventually leads to cell death (Ambekar et al., 2021).

Researchers have observed abnormal auditory function in some patients with neurodegenerative disease syndromes caused by impaired mitochondrial dynamics (Cagalinec et al., 2016; Puusepp et al., 2018; Ham et al., 2019; Choi et al., 2020). Patients with autosomal dominant optic atrophy, induced by mitochondrial fusion gene *OPA1* mutation, often experience hearing loss, which is similar to auditory neuropathy (Ham et al., 2019). Auditory neuropathy is a type of deafness disease characterized by normal OHCs functioning normally, while the IHCs, auditory nerve synapse, and/or auditory nerves are dysfunctional. About 42% of auditory neuropathy is genetically related (Moser and Starr, 2016). The prevalence of auditory neuropathy in UK neonates is 0.039% (46/118925), and this accounts for approximately 6.5% of congenital sensorineural deafness (Smalley and Hole, 2022). As the pathogenic gene of human auditory neuropathy, mitochondrial inner membrane fusion protein OPA1 is an important factor linking abnormal mitochondrial dynamics directly to hearing impairment. The optic nerves of *Opa1*^delTTAG^** mutant mice show an obvious mitochondrial fusion impairment and neuronal death, and hearing function is also significantly impaired (Sarzi et al., 2012). Mutation of another mitochondrial dynamics regulator, *MFN2*, causes the patient to develop Charcot-Marie-Tooth disease, often accompanied by sensorineural deafness (Choi et al., 2020). The mitochondrial fusion and localization in the neurons of *Wfs1* knockout mice are impaired, and about 70% of patients with Wolfram syndrome caused by *WFS1* mutation have sensorineural hearing loss (Cagalinec et al., 2016). These findings suggest that an imbalance in mitochondrial dynamics may be a crucial mechanism of certain hereditary hearing loss.

In addition to the above-mentioned genes involved in the regulation of mitochondrial dynamics, which can lead to hearing loss in humans and animals, the mitochondrial dynamics mechanism is also involved in the occurrence of some hereditary hearing loss. Friedreich’s ataxia (FRDA) is a hereditary neurodegenerative disease caused by mutations in the frataxin (*FXN*) gene encoding the protein frataxin, and sensorineural hearing loss is one of the clinical symptoms of this disease (Rojas et al., 2021). Frataxin is located in mitochondria and plays a key role in the maintenance of mitochondrial function and iron metabolism balance. In 2020, a research team found that after transferring the *FXN* gene into cultured FRDA patient skin biopsy fibroblasts through lentiviral vectors, the mitochondria of the target cells were significantly enlarged. They analyzed the expressions of mitochondrial markers OPA1, DRP1, and MFN1/2 through western blotting, and found that OPA1 expression was significantly elevated in the *FXN* transferred group compared to the control group and that mitochondrial metabolic function was enhanced, thus prolonging cell survival (Agrò and Díaz-Nido, 2020). The involvement of mutations in genes regulating mitochondrial dynamics and their abnormalities in hearing loss models demonstrate the importance of mitochondrial dynamics as a mechanism of hearing loss (Table 1). Thus, mitochondrial dynamics may be a potential drug target for treating or preventing hearing loss.

**TABLE 1:** Mitochondrial dynamics dysfunction induced hearing loss.

Research model	Target gene	Disease	Hearing loss type	Fusion/Fission	Hearing loss/Mitochondrial mechanism	References
Patients with ADOA disease	*OPA1*	Autosomal dominant optic atrophy	Auditory neuropathy	Pro-fusion	Neuronal mitochondria fragmented	Ham et al., 2019
Patients with CMT disease	*MFN2*	Charcot-Marie-Tooth disease	Auditory neuropathy	Pro-fusion	Unmyelinated auditory nerve dysfunction	Choi et al., 2020
C57BL/6J mice	*Drp1*	Age related hearing loss	Age related hearing loss	Pro-fission	Inhibit Drp1, promote the progression of presbycusis	Lin et al., 2019
Fibroblasts from FRDA patient	*FXN*	Friedreich’s ataxia	Auditory neuropathy	Pro-fusion	Iron accumulation and oxidative damage in mitochondria	Rojas et al., 2021
Gene knockout mice	*WFS1*	Wolfram syndrome	Auditory neuropathy	Pro-fusion	Upregulation mitochondrial dysfunction genes	Cagalinec et al., 2016

### Drugs treat hearing loss by modulating mitochondrial dynamics

There are currently no medications approved for the treatment of hearing loss, but some drugs, such as melatonin (Serra et al., 2020) and metformin (Kesici et al., 2018), have demonstrated efficacy in many animal models of hearing loss. Melatonin is a hormone secreted by the pineal gland that targets mitochondria to exert its neuroprotective effects. Its potent antioxidant ability has been validated in various models of hearing loss and tinnitus (Zhou et al., 2021). Recent studies have found that the antioxidant effects of melatonin can be achieved by ameliorating abnormal mitochondrial dynamics (Tan et al., 2016). After adding melatonin to an oxygen/glucose deprivation model constructed in the cultured hippocampal neurons, the ROS production was significantly reduced, the level of mitochondrial fusion proteins MFN2 and OPA1 were enhanced, and the level of mitochondrial fission protein DRP1 was decreased. Therefore, the mitochondria were prone to fusion. These findings imply that melatonin can provide neuroprotective effects through modulating mitochondrial dynamics in damaged hippocampal neurons (Nasoni et al., 2021). In a model of cardiovascular calcification, the mitochondrial fusion protein OPA1 in VSMCs increased, the level of superoxides decreased, and the rate of apoptosis declined after melatonin treatment. The protective effect of melatonin disappeared when the same dose was administered to *OPA1*-knockout vascular smooth muscle. This study confirmed that melatonin reduces cellular oxidative stress by promoting mitochondrial fusion (Tan et al., 2016).

Recently, researchers identified metformin as the top-ranking candidate therapeutic for NIHL when conducting virtual drug screening *via* cochlear single-cell sequencing (Milon et al., 2021). Previous studies (Kesici et al., 2018; Gedik et al., 2020) have shown that metformin protects against noise damage by inhibiting oxidative stress and hair cell apoptosis. However, according to a recently published study of type 2 diabetes mellitus treated with metformin, in the peripheral blood of the metformin treatment group, the expression of leukocyte mitochondrial fusion proteins MFN1/2 and OPA1 increased, the level of mitochondrial fission proteins DRP1 and FIS1 decreased, and the mitochondrial ROS levels were lower than that of the control group. This study linked the cytoprotective effect of metformin to mitochondrial dynamics, and found that the protective effect of metformin may be mediated by promoting mitochondrial fusion (De Marañón et al., 2021). Therefore, the protective effect of melatonin and metformin against noise damage may be achieved by restoring the mitochondrial fusion/fission dynamics.

## Mitochondrial dynamics as an important mechanism for mitochondrial quality control in the inner ear

Mitochondrial quality control refers to the maintenance of homeostasis in cells and organisms by regulating the stability of mitochondrial morphology, quantity, and quality, acting as a crucial barricade mechanism of cells (Pickles et al., 2018). Mitochondrial dynamics are the primary means of regulating mitochondrial quality. Under physiological conditions, mitochondria need to renew and maintain mitochondrial morphology, as well as fuse damaged mitochondria in time, thereby maintaining cellular homeostasis. Under pathological conditions such as stress and hypoxia, an imbalance in mitochondrial dynamics leads to mitochondrial structural damage and dysfunction, which manifests as mitochondrial swelling and cristae re-modeling, decreased membrane potential, impaired ATP production, and excessive ROS and release of pro-apoptotic factors (Pickles et al., 2018). It is well known that the dysregulation of mitochondrial genes and functions is essential for the pathogenesis of hearing loss. The development of options for improving mitochondrial dynamics to repair/prevent the occurrence of hearing loss is an important topic of concern.

Sirtuin-3 (SIRT3), a member of the Sirtuins family, is a deacetylase localized to mitochondria. SIRT3 is the most important mitochondrial deacetylase, which can alter the level of superoxide dismutase 2 (SOD2) acetylation to regulate mitochondrial ROS (Dikalova et al., 2017). More than 90% of the ROS in cells are produced by mitochondria, and acetylation affects the activity of more than a third of mitochondrial proteins. Therefore, SIRT3 plays a vital role in mitochondria and in cells. The hearing protection effect of SIRT3 has been verified in models of age-related hearing loss (Someya et al., 2010), NIHL (Brown et al., 2014), and drug-induced hearing loss (Ding et al., 2021). Glutathione (GSH), sodA2-encoding manganese-containing superoxide dismutase (MnSOD2), and isocitrate dehydrogenase-2 (IDH2) are the most important antioxidant enzymes in cells located in mitochondria. SIRT3 can deacetylate MnSOD2 and IDH2 to upregulate their antioxidant activity and ability to scavenge ROS. Someya’s team found that energy restriction reduced mitochondrial ROS production and increased antioxidant capacity, thus preventing presbycusis in mice. However, under the same energy restriction conditions, the onset of presbycusis was not avoided in *Sirt3*^–/–^ mice. These results show that SIRT3 can enhance mitochondrial antioxidant enzyme activity and accelerate ROS scavenging, there by protecting hearing (Someya et al., 2010). Recent studies have found that *Sirt3*^–/–^ mice develop progressive cardiomyocyte hypertrophy and fibrosis as they age. Abnormally arranged and fragmented mitochondrial cristae have been observed in hypertrophic cardiomyocytes through transmission electron microscopy. Further analysis has shown that the acetylation level of the mitochondrial fusion protein OPA1 was elevated in aging mice. After injecting the *Opa1* gene into the tail vein of *Sirt3*^–/–^ mice by adeno-associated virus (AAV), myocardial mitochondrial cristae structure, mitochondrial antioxidant capacity, and ATP synthesis were restored, thereby delaying cardiac aging (Benigni et al., 2019). Therefore, the mitochondrial SIRT3/OPA1 regulatory axis is a critical mechanism for cellular anti-oxidation and the prevention of aging. We suggest that suppressing the abnormal mitochondrial oxidative stress response by modulating mitochondrial dynamics in mouse cochlear hair cells may protect auditory function (Lin et al., 2019).

## Conclusion: problems and prospects

Based on all the literature we discussed above on hearing loss associated with abnormal mitochondrial dynamics, mitochondrial dynamics tend to be in the state of fission under stress conditions such as noise and aging, and excessive fission can lead to insufficient mitochondrial ATP synthesis and excessive ROS production, which results in the death of auditory cells. Promoting mitochondrial fusion can supplement mtDNA in mitochondria, increase ATP synthesis, and reduce ROS, thereby preventing cell death. The above literature suggests that mitochondrial dynamics are a key mechanism for mitochondrial quality control and cellular anti-oxidation, which has been validated in many models of nerve injury and neurodegeneration (Yang et al., 2021). However, in the field of inner ear and hearing loss models, most studies continue to focus on mitochondrial functions such as the mitochondrial apoptosis pathway, mitophagy, ROS release, and ATP synthesis, and the mitochondrial dynamics that govern mitochondrial function have not yet been investigated in depth. For example, the SGNs have long-distance axonal projections, and the mitochondrial dynamics regulation in the network connection between axonal mitochondria and soma mitochondria is more compelling (Wang et al., 2021). Thus, mitochondrial dynamics, as an important mechanism for regulating mitochondrial function, are worthy of further exploration by researchers.

## Author contributions

MX made substantial contributions to the design of the work and approved the final version of this article. TZ, XS, BY, KC, AZ, DG, YP, RD, and HH participated in the conception and design of the study, collected the literature, and wrote the manuscript. All authors agreed to be accountable for all aspects of the work in ensuring that questions related to the accuracy or integrity of any part of the work are appropriately investigated and resolved and contributed to the article and approved the submitted version.
